# A Pan‐Hepatic T‐Cell Atlas Identifies Disease‐Specific Immune Dysfunction and Therapeutic Targets for Immunotherapy

**DOI:** 10.1002/mco2.71146

**Published:** 2026-11-08

**Authors:** Si‐Yuan Chen, You‐Yuan Wang, Shuang‐Nan Zhou, Ming‐Ju Zhou, Xia Li, Wei‐Zhe Li, Meng‐Meng Zhu, Yu‐Xuan Yang, Ying Sun, Peiyao Fan, Jing Li, Xing Fan, Ruonan Xu, Yan‐Mei Jiao, Jin‐Wen Song, Cheng Zhen, Ming Shi, Chao Zhang, Fu‐Sheng Wang

**Affiliations:** ^1^ Senior Department of Infectious Diseases Chinese PLA General Hospital Beijing China; ^2^ National Clinical Research Center for Infectious Diseases Beijing China; ^3^ The Second School of Clinical Medicine Southern Medical University Guangzhou China

**Keywords:** hepatic diseases, single‐cell T‐cell atlas, exhausted CD8^+^ T cells, regulatory CD4^+^ T cells, cellular and molecular targets

## Abstract

Single‐cell transcriptomics has greatly advanced insight into hepatic immunity and immunotherapy. However, the absence of an integrated atlas spanning multiple hepatic diseases limits systematic comparisons of immune heterogeneity and dynamics. Here, we established a Pan‐HEpatic single‐cell Atlas of T cells (Pheat), covering 139 donors, 255 blood and liver samples spanning chronic hepatitis (chronic hepatitis B/C [CHB/CHC], alcohol‐associated hepatitis, metabolic dysfunction‐associated steatohepatitis), cirrhosis, hepatocellular carcinoma (HCC), and healthy controls (HC), and delineated profound population‐, tissue‐, and disease‐level heterogeneity of 624,215 T cells, with emphasis on CD8^+^ exhausted T cells (Tex) and CD4^+^ regulatory T cells (Treg). Within Tex, intrahepatic precursor Tex (Tex^pre^) were depleted in CHB compared with healthy controls and other hepatic diseases. Disease‐specific checkpoint dominance (4‐1BB in CHB, TIGIT in HCC) in Tex^pre^ was identified and experimentally validated. Panobinostat was predicted and validated to reinvigorate Tex^pre^ in CHB patients. In parallel, effector Treg (eTreg) accumulated in diseased livers and exhibited divergent IL‐2–STAT5 signaling—hyperactive in CHB and HCC, suppressed in cirrhosis—indicating distinct immunosuppressive status. Lapatinib/olopatadine enhanced Treg suppressive markers in cirrhosis. Pheat provides a high‐resolution atlas of hepatic T cells, revealing disease‐specific immune heterogeneity and regulatory programs that inform cellular and molecular targets for potential immunotherapy.

## Graphical abstract

A Pan‐HEpatic single‐cell Atlas of T cells (Pheat) was constructed covering chronic hepatitis, cirrhosis, hepatocellular carcinoma (HCC), and healthy controls. CD8^+^ exhausted T cells (Tex) and CD4^+^ regulatory T cells (Treg) were delineated profound through decoding molecular and cellular variance, immune checkpoint networks, pathway analysis, and drug prediction, supported by flow cytometry, multiplex immunofluorescence, and *ex vivo* functional assays.

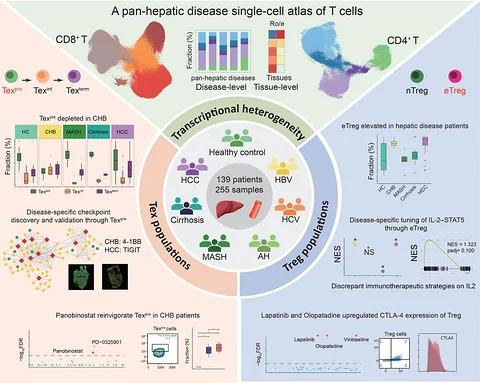

## 1 Introduction

Hepatic diseases, including chronic hepatitis, cirrhosis, and HCC, collectively cause over two million deaths annually, representing approximately 4% of all global mortality [1]. Many of these conditions arise from progressive pathological cascades, where risk factors like viral infection, alcohol abuse, and metabolic dysfunction–associated steatotic liver disease trigger chronic liver inflammation, fibrosis, and eventual malignancy [2]. Central to this progression is the hepatic immune system, where T cells play a dual role: they are essential for viral clearance and tumor surveillance, but can also drive immunopathology. Understanding and manipulating these T‐cell responses is therefore pivotal for therapeutic innovation.

T cells, particularly CD4^+^ helper and CD8^+^ cytotoxic subsets, are central to hepatic adaptive immunity, playing crucial roles in antigen recognition, antiviral responses, immune surveillance, and elimination of diseased hepatocytes [3, 4, 5]. In chronic hepatitis and HCC, however, persistent antigen stimulation drives CD8^+^ T cells into a dysfunctional state known as exhaustion (Tex) [6, 7]. This is not a single state but a continuum, ranging from Tex^pre^ cells, which retain proliferative potential and responsiveness to immunotherapy, to terminally Tex (Tex^term^), which are functionally inert [8, 9, 10, 11]. In parallel, immunosuppressive Tregs accumulate in diseased liver tissue, actively suppressing antiviral and anti‐tumor responses [12, 13, 14]. eTregs, in particular, are enriched in these microenvironments and are characterized by high expression of inhibitory molecules, further crippling effective immunity [15]. This dual challenge of T‐cell exhaustion and Treg‐mediated suppression forms a major barrier to resolving chronic hepatic disease.

Current immunotherapeutic strategies, most notably immune checkpoint blockade (ICB), are designed to overcome these barriers. Antibodies targeting co‐inhibitory receptors like PD‐1 and CTLA‐4 can reinvigorate Tex and curb the suppressive function of Tregs [16, 17, 18]. In HCC, PD‐1 inhibitors such as nivolumab and pembrolizumab—alone or in combination with CTLA‐4 blockade (e.g., ipilimumab) have led to durable responses in a subset of patients and have received US Food and Drug Administration (FDA) approval [19, 20, 21]. Similarly, in CHB, PD‐1 blockade can partially restore antiviral immunity [22, 23, 24]. However, the overall efficacy of these therapies remains suboptimal, with highly heterogeneous response rates and inconsistent benefits across different disease etiologies [25, 26, 27]. One major reason is that T‐cell dysfunction is context‐dependent: the specific states of Tex and Treg differ substantially between chronic hepatitis, cirrhosis, and HCC.

While pan‐cancer single‐cell atlas studies of T [28, 29, 30], B [31], NK [32, 33], and myeloid cells [34] have successfully mapped immune states across various tumors, meanwhile, a comprehensive map defining the normal functions of diverse liver cell types and their spatial relationships has facilitated understanding of liver immune microenvironment [35]; however, the diseased hepatic T‐cell landscape remains fragmented. A systematic, cross‐disease comparison is urgently needed to understand how distinct liver pathologies shape T‐cell dysfunction, a gap that currently hinders the development of precise immunotherapies. Here, we integrated large‐scale single‐cell RNA‐Sequencing (scRNA‐Seq) data from patients with six different hepatic diseases and healthy donors to construct Pheat, a pan‐hepatic single‐cell atlas of T cells, and decoded molecular and cellular variance, immune checkpoint networks, pathway analysis, and drug prediction, supported by flow cytometry, multiplex immunofluorescence, and *ex vivo* functional assays. This resource enables systematic mapping of canonical and unconventional T‐cell lineages and, importantly, reveals both shared and disease‐specific Tex and Treg programs. By uncovering shared and disease‐specific immune states, Pheat provides a valuable resource to inform the development of precision immunotherapies in hepatic diseases.

## 2 Results

### 2.1 Construction of a Pan‐Hepatic T‐Cell Atlas (Pheat)

To create a comprehensive resource for studying T‐cell immunity in hepatic disease, we curated the Pheat by integrating 139 donors, 255 blood and liver scRNA‐Seq samples from 12 distinct studies (Figure 1A; Table S1). This dataset encompasses a wide range of conditions, including patients with HBV infection, HCV infection, alcohol‐associated hepatitis (AH), metabolic dysfunction‐associated steatohepatitis (MASH), cirrhosis, HCC, and HC, covering diverse demographics, multiple etiologies and disease states (Figure 1A, B).

**FIGURE 1:**
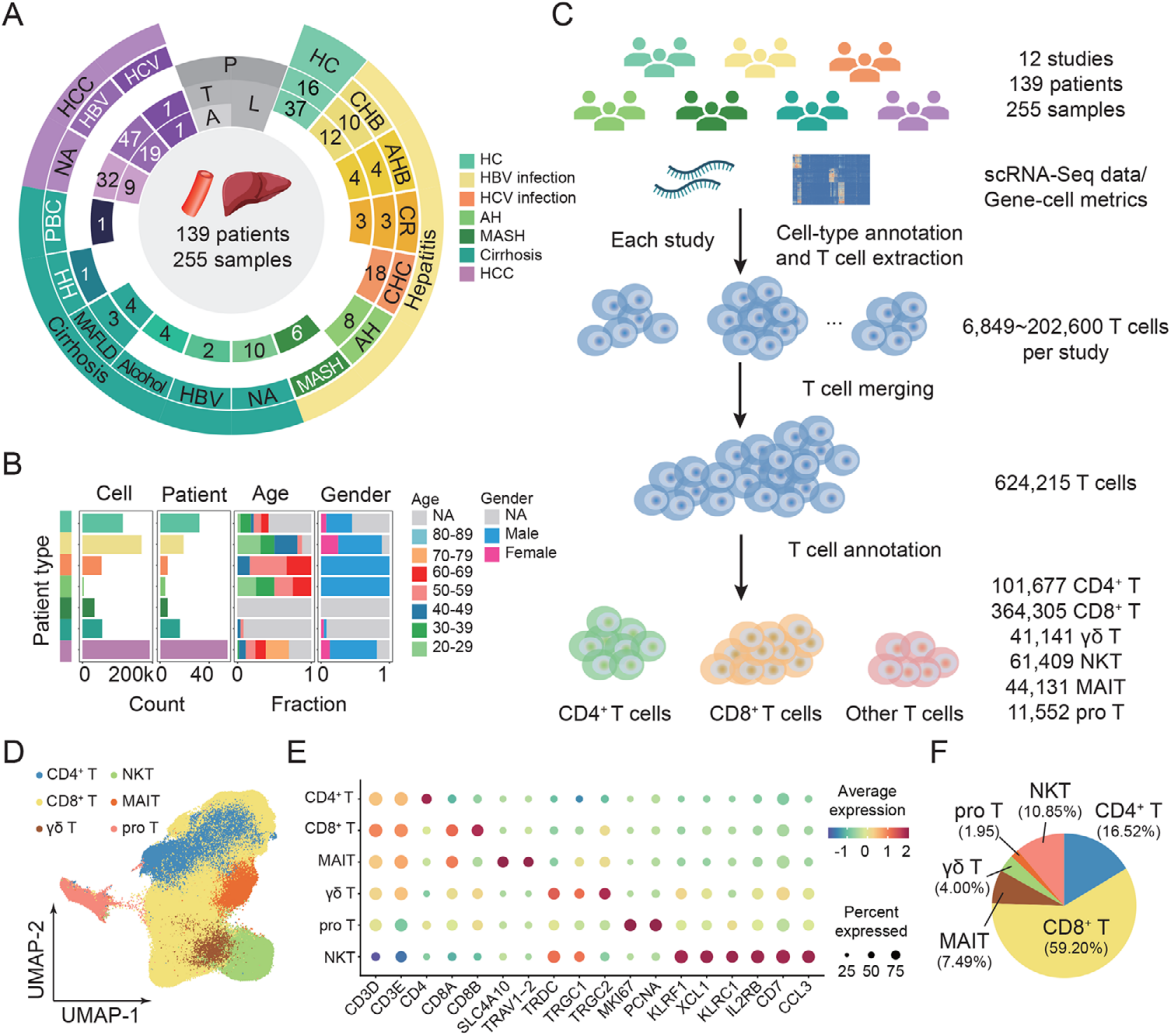
**Overview of the Pheat: data collection and major T‐cell annotation**. (A) Disease and tissue information of scRNA‐Seq data in Pheat. Arabic numerals are the number of samples. The outermost ring indicates different hepatic diseases. The second ring represents the corresponding disease states or etiologies. The third and fourth rings show the numbers of peripheral blood and liver tissue samples, respectively. The innermost ring indicates the number of adjacent non‐tumor tissue samples from patients with HCC. Abbreviations: P, peripheral blood mononuclear cells (PBMC); L, liver tissue; T, tumor tissue; A, adjacent tumor tissue; HC, healthy control; CHB, chronic hepatitis B; AHB, acute hepatitis B; CR, chronic resolved for CHB; CHC, chronic hepatitis C; AH, alcohol‐associated hepatitis; MASH, metabolic dysfunction‐associated steatohepatitis; NA, not available; HBV, hepatitis B virus; MAFLD, metabolic dysfunction–associated steatotic liver disease; HH, hereditary haemochromatosis; PBC, primary biliary cholangitis; HCV, hepatitis C virus. Detailed information is provided in Table S1. (B) Summary statistics for the number of cells and patients, and the distribution of ages and gender in individuals with differential hepatic disease and HC donors. (C) Schematic depicting the process of the Pheat construction. (D) UMAP view of six major T‐cell clusters. (E) Marker gene expression across T‐cell clusters. The size of the bubble is proportional of cells expressing a gene and the color key is proportional to the scaled gene expression. (F) Cellular frequencies of the major T‐cell types in Pheat.

After stringent quality control, doublet and batch effect removal, we isolated 624,215 high‐quality T cells. Through unsupervised clustering and annotation based on canonical markers, we resolved these cells into six major lineages: CD4^+^ T, CD8^+^ T, γδ T, natural killer T (NK T), mucosal‐associated invariant T (MAIT) and proliferative T (pro T) cells (Figures 1C, E and S1).

To facilitate community‐wide exploration of this rich dataset, we developed an interactive web portal (Figure S2), providing open access to gene expression data, cell annotations, and visualization tools.

### 2.2 Transcriptional Heterogeneity of T Cells Deposited in Pheat

Within Pheat, CD8^+^ T cells were the most abundant lineage, prompting a detailed sub‐clustering analysis to further characterize their heterogeneity (Figure 1F). We identified five distinct transcriptional states present across all diseases through canonical genes and pathways, albeit in varying proportions (Figures 2A and S3A): (1) a naive‐like cluster (Tn, *IL7R*, *CCR7*, *SELL*); (2) two distinct effector clusters (Effector‐KLRG1, Teff^KLRG1^; Effector‐CD16, Teff^CD16^); (3) an interferon‐stimulated gene (ISG) signature cluster (T_ISG_, *IFIT1*, *STAT1*, *MX1*); and (4) an exhausted cluster (Tex) defined by high expression of inhibitory receptors, such as *PDCD1*, *CTLA4*, and *TIGIT* (Figures 2B and S3B; Table S2).

**FIGURE 2:**
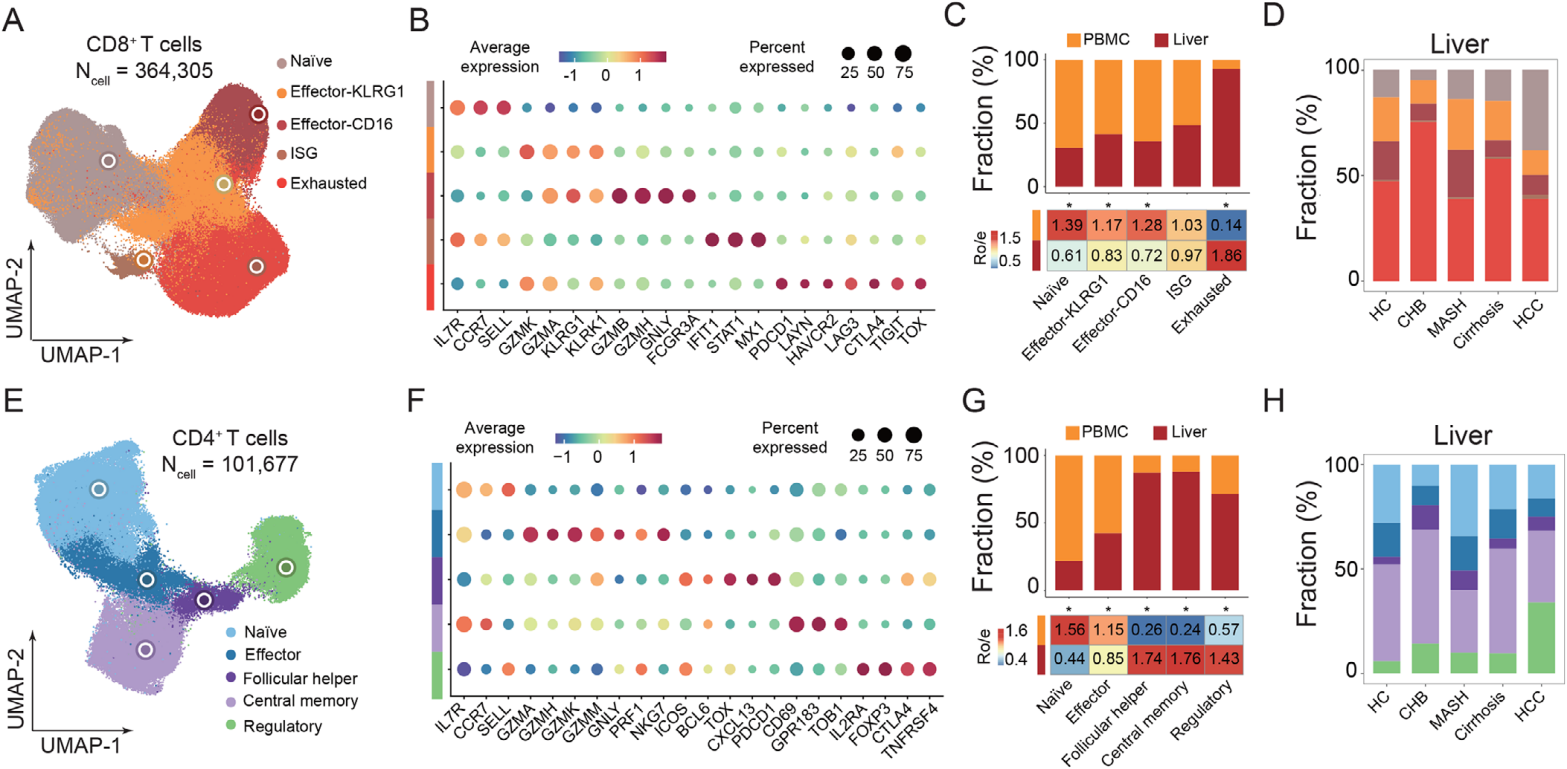
**Transcriptional diversity of CD8^+^ T and CD4^+^ T cells**. (A) UMAP view of five CD8^+^ T‐cell clusters. (B) Bubble plot showing marker gene expression across differential CD8^+^ T clusters. (C) Distribution of CD8^+^ T clusters across PBMC and liver tissue. Bar plot (upper) showing the relative proportion of cells for each CD8^+^ T‐cell type in PBMC and liver tissue. Heat map (lower) displaying tissue prevalence estimated by Ro/e. Significant levels were marked by asterisks as *p*‐adjusted less than 0.05. (D) Cellular proportion of five CD8^+^ T‐cell states across individuals with different hepatic diseases and HC donors in the liver tissue. (E) UMAP view of five CD4^+^ T‐cell subsets. (F) Bubble plot showing marker gene expression across differential CD4^+^ T clusters. (G) Distribution of CD4^+^ T clusters across PBMC and liver tissue. Bar plot (upper) showing the relative proportion of cells for each CD4^+^ T‐cell type in PBMC and liver tissue. Heat map (lower) displaying tissue prevalence estimated by Ro/e. (H) Cellular proportion of five CD4^+^ T‐cell states across individuals with differential hepatic diseases and HC donors in liver tissue.

These CD8^+^ T‐cell subsets displayed clear tissue compartmentalization. Using Ro/e analysis, we confirmed that naïve and effector subsets were significantly enriched in peripheral blood (PBMC), whereas the Tex subset was predominantly localized to the liver (Figures 2C and S3C, D). Notably, among all diseases, the frequency of intrahepatic Tex cells was highest in patients with CHB (Figures 2D and S3E).

Similarly, we resolved the CD4^+^ T‐cell population into five canonical subsets: Naïve (Tn, *IL7R*, *CCR7*, *SELL*), Effector (Teff, *GZMA*, *GZMH*, *GZMK*, *GZMM*), Follicular helper (Tfh, *ICOS*, *BCL6*, *TOX*, *CXCL13*, *PDCD1*), Central memory (Tcm, *CD69*, *GPR183*, *TOB1*), and Regulatory (Treg, *IL2RA*, *FOXP3*, *CTLA4*, *TNFRSF4*) cells (Figures 2E, F and S4A, B; Table S2). Consistent with the CD8^+^ T‐cell compartment, CD4^+^ T‐cell subsets also showed significant tissue‐specific enrichment, with Tfh, Tcm, and Treg cells being more abundant in the liver (Figures 2G and S4C, D). In the intrahepatic environment, Treg cells showed an obvious heterogeneity across diseases, with the highest frequency observed in HCC patients (Figures 2H and S4D, E).

We also characterized pro T cells and unconventional T‐cell populations, which likewise exhibited disease‐ and tissue‐specific distributions. Notably, we observed a heterogeneous enrichment of pro_CD8‐CCL4 in liver tissue, in parallel, a dominant cellular frequency of NKT, especially NKT‐TIGIT, whereas a low frequency of MAIT in the peripheral blood of AH (Figure S5).

### 2.3 Dysfunctional Trajectories of the Tex Population in Hepatic Diseases

As T‐cell exhaustion accompanies disease progression and constitutes the direct target of ICB, we performed an in‐depth analysis of the Tex population. Iterative clustering of Tex cells revealed three distinct subsets: Tex^pre^ (*TCF7* ^+^ *CD69^+^HAVCR2* ^−^), retaining stem‐like and effector potential; intermediate Tex (Tex^int^) (*SLAMF6* ^−^ *CD69* ^−^ *TBX21* ^+^), a transitional state with proliferative properties; Tex^term^ (*TCF7* ^−^ *HAVCR2* ^+^), representing an irreversibly dysfunctional state (Figures 3A, B and S6A). These results were consistent with the progressive model of T‐cell exhaustion [36, 37, 38]. Functional enrichment analysis confirmed this hierarchy: Tex^pre^ cells were enriched for pathways related to stemness and activation, while Tex^term^ cells exhibited the highest signature of dysfunction and immune suppression (Figures 3C‐E and S6B; Tables S3 and S4).

**FIGURE 3:**
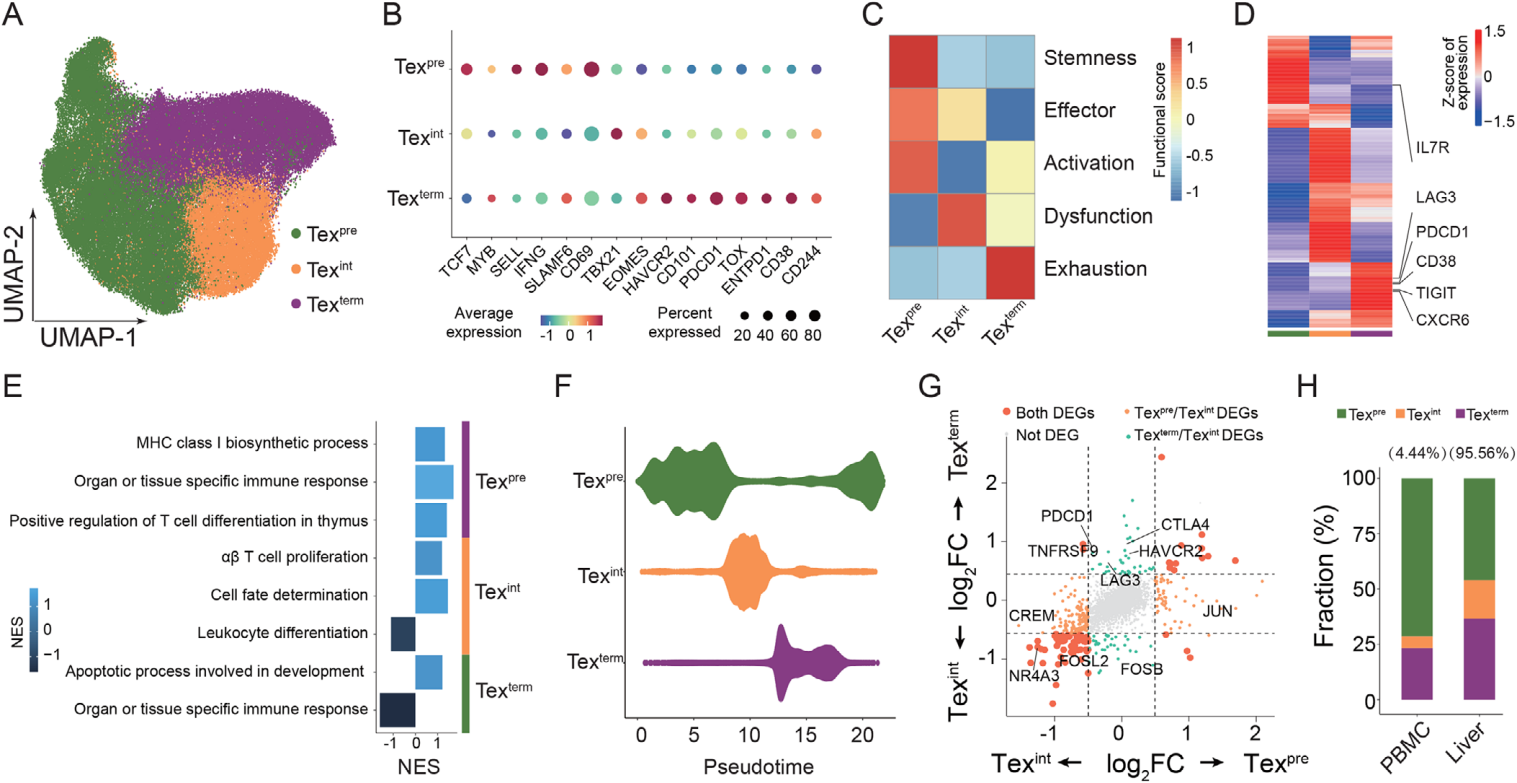
**Immunological characteristics of the Tex population**. (A) UMAP view of three Tex subpopulations, including Tex^pre^, Tex^int^, and Tex^term^. (B) Bubble plot showing marker gene expression across differential Tex subpopulations. (C) Expression of five gene signatures, including stemness, effector, activation, dysfunction, and exhaustion (as listed in Table S3) across differential Tex clusters. (D) Heat map showing the expression of DEGs across three Tex subpopulations. (E) Functional enrichment across three Tex subpopulations using differential expression analysis results. Abbreviations: NES, Normalized enrichment score. (F) Pseudotime analysis of cell states across Tex subpopulations. (G) Fold change (FC) of differential expression analysis estimated between two neighboring Tex subpopulations. (H) Bar plot showing the relative proportion of cells for each Tex cell type in PBMC and liver tissue.

Pseudotime analysis predicted a development trajectory from Tex^pre^ cluster, through Tex^int^ to Tex^term^ subpopulation (Figures 3F and S7A), consistent with established models of progressive T‐cell exhaustion [8, 9, 36, 37, 38, 39]. Differential abundance analysis showed that Tex^pre^ cells were enriched in low HBsAg and HBV DNA levels, whereas the enrichment status of low‐level HBV antigen load was decreased in Tex^int^ and Tex^term^ (Figure S7B). T‐cell receptor (TCR) analysis upon intrahepatic Tex cells in CHB patients showed Tex^pre^ cells were predominantly composed of singleton and low‐frequency clonotypes, whereas Tex^int^ and particularly Tex^term^ cells showed progressively clonal expansion (Figure S7C‐E). STARTRAC transition scores among the three Tex subpopulations were also consistent with the developmental trajectory (Figure S7F). A series of differentially expressed genes (DEGs) estimated between two neighboring Tex states were decoded transcriptional factors triggering the state shift of Tex, including *JUN*, *NR4A3*, *CREM* during the process from Tex^pre^ to Tex^int^, and *FOSB* and *FOSL2* during Tex^int^ to Tex^term^ (Figure 3G; Table S5). SCENIC analysis identified a regulatory network involving *LEF1*, *TBX21*, *EOMES*, *BATF3*, *EGR2*, and *NF‐κB* family members across the Tex differentiation continuum (Figure S7G).

We next examined how these exhaustion states are distributed across tissues. Intrahepatic Tex cells were consistently more frequent than in PBMC (Figures 2C, 3H and S2C, D). At the subpopulation level, Tex^int^ and Tex^term^ clusters were increased in the liver, whereas Tex^pre^ cells showed the opposite trend (Figure 3H).

### 2.4 Unveiling Disease‐Specific Signatures and Immunotherapeutic Targets With the Intrahepatic Tex^pre^ Subpopulation

The established role of Tex^pre^ cells as the primary responders to ICB therapy [40] prompts us to further unveil cellular and molecular features of the intrahepatic Tex^pre^. Tex^pre^ cells were a predominant intrahepatic Tex subset across hepatic diseases, but were significantly reduced in CHB (Figures 4A and S8). A similar shift was observed along the HBV disease trajectory, from CHB to HBV^+^ cirrhosis and HBV^+^ HCC (Figure S9A). In HCC, Tex^pre^ cluster was more abundant in tumor tissue of HBV^−^ cases than HBV^+^ cases (Figure S10A). The depletion of Tex^pre^ in CHB may be attributable to the heavy HBV antigen burden, which accelerates their transition toward more differentiated exhaustion states and diminishes the stem‐like pool [10]. At the molecular level, we identified several disease‐associated signatures within the Tex^pre^ cluster, such as *MAF* in CHB, *CCL20* in HBV^+^ cirrhosis, and heat shock proteins (e.g., *HSPA1B*, *HSPA6*) in HBV^+^ HCC (Table S6). Collectively, these findings highlight that Tex^pre^ cells not only reflect the distinct immunological pressures imposed by disease etiology and stage, but also capture disease‐specific molecular imprints associated with viral control, fibrotic remodeling, and tumor progression.

**FIGURE 4:**
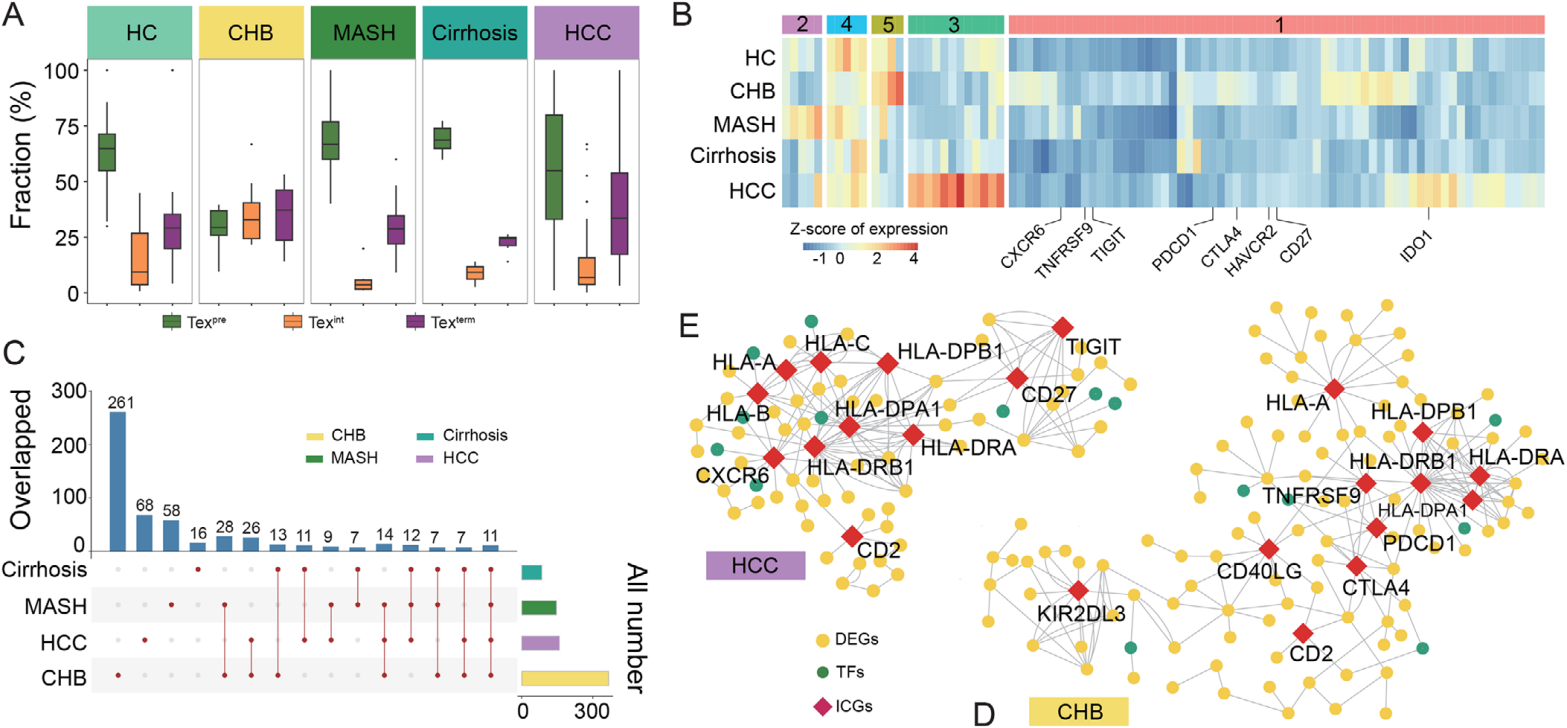
**Disease‐specific signature and immunotherapeutic target identification with the intrahepatic Tex^pre^ subpopulation**. (A) Box plot showing the cellular proportion of the three types of intrahepatic Tex subpopulations across individuals with differential hepatic diseases and HC donors. (B) Heat map of expression of ICGs in the intrahepatic Tex^pre^ subpopulation across individuals with differential hepatic diseases and HC donors through pseudo‐bulk analysis. (C) The number of DEGs in the intrahepatic Tex^pre^ subpopulation across individuals with differential hepatic diseases compared to HC donors. (D, E) Co‐expression networks constructed by Cytoscape using DEGs and ICGs in the intrahepatic Tex^pre^ subpopulation from CHB patients (D) and HCC patients (E).

Next, we focused on the intrahepatic Tex^pre^ to identify novel, disease‐specific immunotherapeutic targets. We analyzed the expression of 93 immune checkpoint genes (ICGs) [41, 42]. A pseudobulk profile revealed that the majority (72.04%, 67/93) of ICGs showed unbiased gene expression of ICGs among different hepatic diseases (Figure 4B; Table S7). To identify disease‐specific ICGs, we first depicted the gene variant on expression levels by comparing intrahepatic Tex^pre^ of each disease to HC donors (Figure 4C; Table S8). Then, disease‐specific co‐expression networks were constructed through the abundance of ICGs and DEGs (Figures 4D, E and S11A, B). In the CHB patient‐network, several hub ICGs were identified based on the correlation status of ICG‐DEG pairs, including *PDCD1*, *TNFRSF9*, *CTLA4*, *CD40LG*, *CD2*, *KIR2DL3*, and HLA‐related genes, which might be potential immunotherapeutic targets for CHB patients (Figure 4D). Of these, *PDCD1* and *CTLA4* have been proven to be safe and effective treatments through reinvigorating Tex cells [16, 17, 18, 22, 43]. *TNFRSF9* (4‐1BB) shows a strong potential to rejuvenate dysfunctional CD8^+^ T cells in HBeAg^+^ chronic patients [42]. Several hub ICGs, including *CXCR6*, *CD2*, *CD27*, *TIGIT*, and some HLA‐related genes, were regarded as preferential immunotherapeutic targets in HCC (Figure 4E). Of these, *CXCR6* was reported as a signature for chronically activated T cells, which can promote CD8^+^ T‐cell survival and control tumor growth in mouse models [28].

To validate these computational predictions, we performed multiplex immunofluorescence on patient liver tissues (Figures 5A and S12A). We confirmed a marked enrichment of intrahepatic Tex^pre^ (CD8^+^PD‐1^+^TCF1^+^) cells in HCC tumors relative to CHB (Figure 5B), consistent with our scRNA‐Seq data (Figure 4A). Crucially, we verified that Tex^pre^ cells showed higher 4‐1BB expression in CHB, while TIGIT was elevated in HCC (Figure 5C), providing evidence for these disease‐specific therapeutic targets.

**FIGURE 5:**
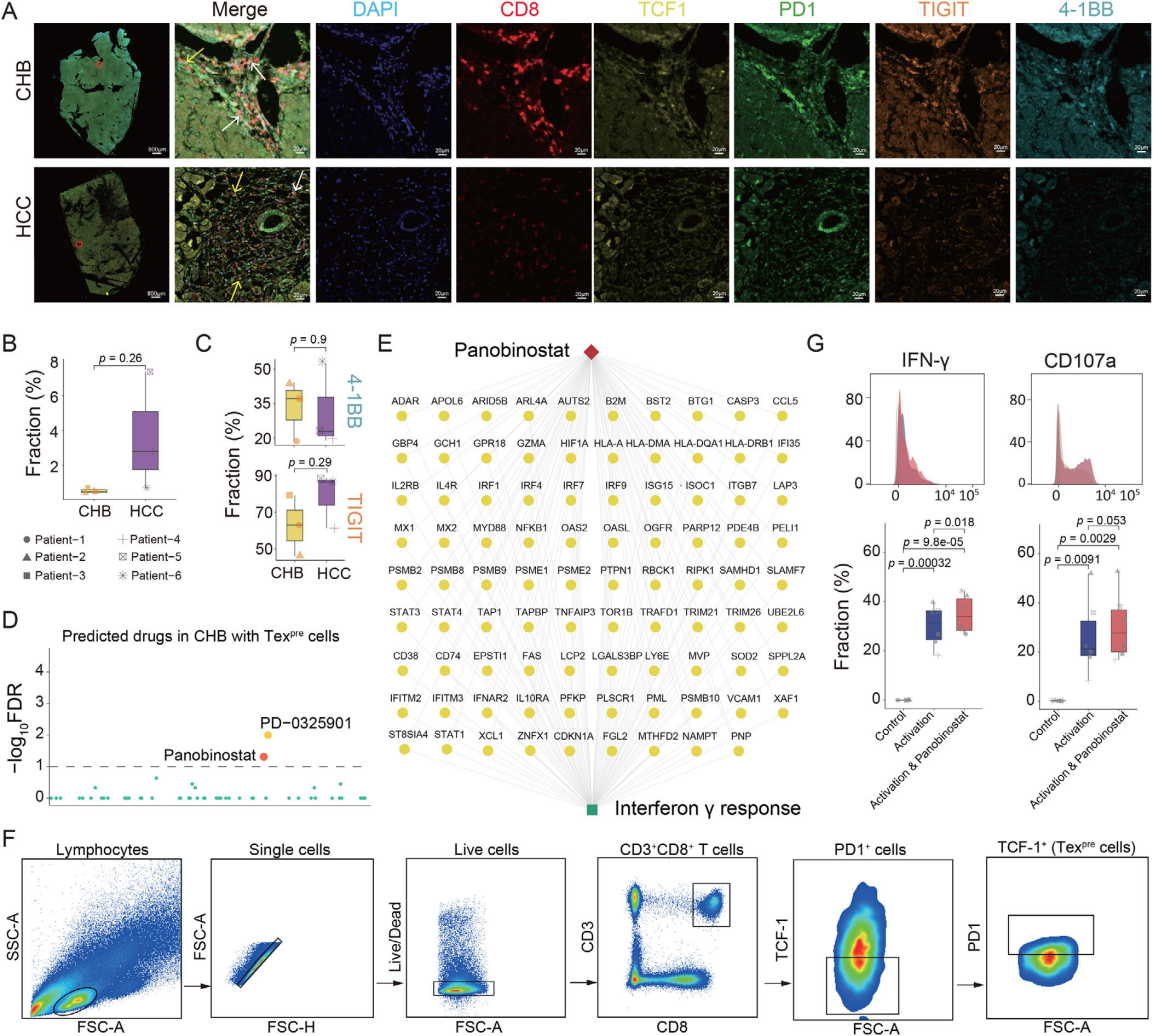
**Phenotypic characterization of intrahepatic Tex^pre^ cells and identification of drug repurposing on intrahepatic Tex^pre^ subpopulation**. (A) Multiplexed immunofluorescent staining of the Tex^pre^ cells marker panels (DAPI‐blue, CD8‐red, TCF1‐yellow, PD1‐green, TIGIT‐orange and 4‐1BB‐light blue) of liver tissue in the CHB and HCC groups (40×, scale bars = 20 µm). The white arrows marked the Tex^pre^ cells expressing 4‐1BB, and the yellow arrows marked the Tex^pre^ cells expressing TIGIT. (B) The percentage of Tex^pre^ cells in total CD8^+^ T cells within the liver tissue of the CHB (*n* = 3) and HCC (*n* = 3) groups. (C) Quantification of patients with Tex^pre^ cells expressing 4‐1BB and TIGIT between the CHB and HCC groups. The CHB and HCC groups each contained three biological replicates (*n* = 3). In the CHB group, the mean number of view fields was 238 (224, 263, 228), and the mean total cell count was 195,628 (197,555, 235,890, 153,439). In the HCC group, the mean number of microscopic fields was 300 (183, 259, 460), and the mean total cell count was 318,182 (207,822, 313,760, 432,966). (D) Predicted drugs and compounds to reverse the expression patterns based on the intrahepatic Tex^pre^ subpopulation in CHB individuals. (E) Panobinostat treatment impacts multiple genes enriched in the interferon γ response pathway. (F) The gating strategy for Tex^pre^ cells. (G) Representative IFN‐γ (left) and CD107a (right) expression in Tex^pre^ cells using PBMC from CHB patients (*n* = 6) of unstimulated (gray), anti‐CD3/CD28 and PMA/Iono stimulated (blue), and 1 nM Panobinostat intervention (red).

We tried to further probe potential immunotherapeutic targets for different hepatic diseases through intrahepatic Tex^pre^ subpopulations (Figures 5D and S12B) with ASGARD [44], a computational tool that estimates drug efficacy using the L1000 drug response dataset [45]. Through making use of DEGs estimated by comparing different hepatic disease patients to HC donors in the intrahepatic Tex^pre^ subpopulation (Figure 4C; Table S9), Panobinostat, a broad‐spectrum histone deacetylase (HDAC) inhibitor, was predicted as a candidate drug in CHB patients to significantly reverse gene expression patterns using 367 DEGs as inputs (Figure S12C). Gene enrichment analysis of the DEGs from the Tex^pre^ population of CHB patients highlighted several biological pathways, particularly the interferon γ response pathway [46] (Figures 5E and S12C; Table S10), which was implicated in acute hepatitis B (AHB) and chronic resolved (CR) individuals, underscoring its critical role in the cure of HBV infection (Figure S12D‐G; Table S10). To experimentally validate the predicted effect of Panobinostat, we assessed its impact on the antiviral function using PBMC from CHB patients (Table S11). As predicted, Panobinostat treatment significantly enhanced the production of IFN‐γ and CD107a in the Tex^pre^ subpopulation (Figures 5F, G and S12H), while failing to restore IFN‐γ expression in Tex^term^ cells (Figure S12I), indicating a restoration of effector activity. These results provide functional evidence that Panobinostat may reinvigorate Tex^pre^ cells via activation of the interferon‐γ pathway, supporting its potential as an immune‐modulating candidate in CHB.

### 2.5 Suppressive Profile of Treg Population in Hepatic Diseases

To explore the regulatory arm of hepatic immunity, we turned our focus to the Treg compartment, a major driver of immunosuppression. After re‐clustering, two transcriptionally distinct subsets were identified, namely naïve Treg (nTreg, *PTPRC* ^high^ *FOXP3* ^low^) and eTreg (*PTPRC* ^low^ *FOXP3* ^high^) subpopulations (Figure S13A, B) [15]. Tissue enrichment analysis revealed that eTreg preferentially localized in the liver, while nTreg were predominantly found in peripheral blood (Figure 6A). Given that distinct molecular programs underlie Treg suppressive function, we compared the expression of immunoregulatory genes between the two subsets, with eTregs exhibiting markedly higher levels of signals (such as *TIGIT*, *JUNB*, *CTLA4*, *ICOS*) linked to suppressive function, indicating that eTreg represents the dominant immunoregulatory population within hepatic tissue (Figure 6B) [47].

**FIGURE 6:**
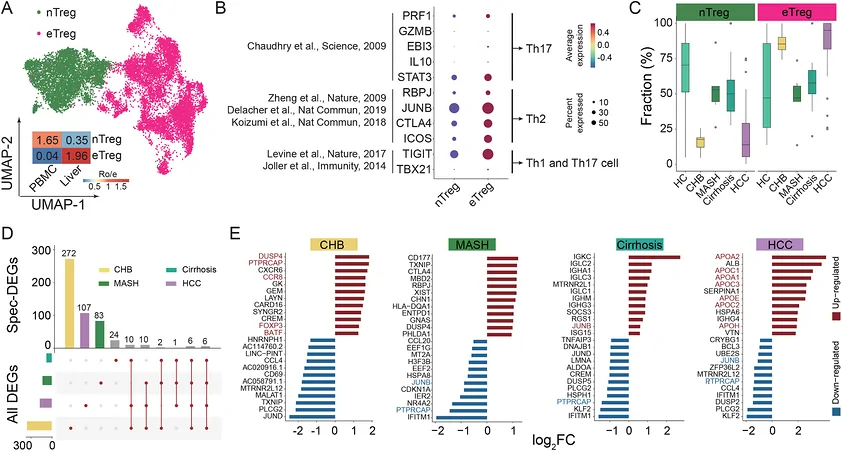
**Immunological characteristics of the Treg cell population**. (A) UMAP view of two Treg subpopulations, including nTreg and eTreg. Heat map displaying tissue prevalence of two Treg subpopulations in PBMC and the liver tissue estimated by Ro/e. (B) Bubble plot showing expression of representative genes associated with Treg function and regulation collected from [47] in two Treg subpopulations. (C) Cellular proportion of two Treg subpopulations among individuals with differential hepatic diseases and HC donors. (D) The number of DEGs is estimated by comparing the eTreg from individuals with differential hepatic diseases to HC donors. (E) The most variant DEGs from (D).

We next explored how eTreg varies across hepatic diseases. The proportion of intrahepatic eTreg cells was consistently elevated in hepatic disease patients compared to HC donors (Figure 6C). Differential expression analysis revealed disease‐specific transcriptional programs within eTregs (Figure 6D; Table S12). CHB patients showed a higher expression abundance in intrahepatic eTreg cells than HC donors for several Treg‐related signatures, such as *DUSP4*, *CCR8*, *FOXP3*, and *BATF* (Figure 6E). HCC‐derived eTregs showed metabolic reprogramming characterized by upregulation of multiple apolipoproteins (*APOA1/2*, *APOC1/2/3*, and *APOH*), indicating potential lipid‐associated adaptation to the tumor microenvironment (Figure 6E). Several shared DEGs presented in different hepatic diseases compared with HC donors, such as *JUNB* and *PTPRCAP* (Figure 6E).

### 2.6 Divergent IL‐2–STAT5 Signaling and Drug Repurposing on Intrahepatic eTreg Subpopulation

To characterize disease‐specific regulatory mechanisms underlying intrahepatic eTreg activation, functional pathways related to transcriptional variance of genes were further investigated, while pathways related to Treg activation, such as Myc targets, TNFα signaling via NFkB, mTOR signaling, and PI3K Akt mTOR signaling, were commonly enriched across hepatic diseases (Figures 7A and S14; Table S13). It is noted that IL‐2–STAT5 signaling is enriched in all hepatic diseases, with directionality differing in CHB, cirrhosis, and HCC (Figure 7B, C). CHB and HCC patients exhibited a significantly higher expression abundance of genes enriched in IL‐2–STAT5 signaling, whereas cirrhosis patients showed an inverse status. As the role of IL‐2 in modulating the Treg suppressive function [48] and as a therapeutic target for several diseases [49, 50], these results indicated disease‐specific tuning of IL‐2–STAT5 signaling. In addition, by extracting the IL‐2–STAT5 signaling‐driven genes for each disease, we observed a series of disease‐specific genes, such as *NFIL3* for MASH, *BHLHE40* for cirrhosis, *IFITM3*, *SPP1*, and *SOCS2* for HCC tumor tissue (Figure 7D; Table S14). We also identified several ICGs with differential expression patterns along the development of CHB and HCC with different causes, such as *ICOS*, *TIGIT*, and HLA‐related genes (Figures S15 and S16), highlighting potential immunotherapeutic targets in these hepatic contexts.

**FIGURE 7:**
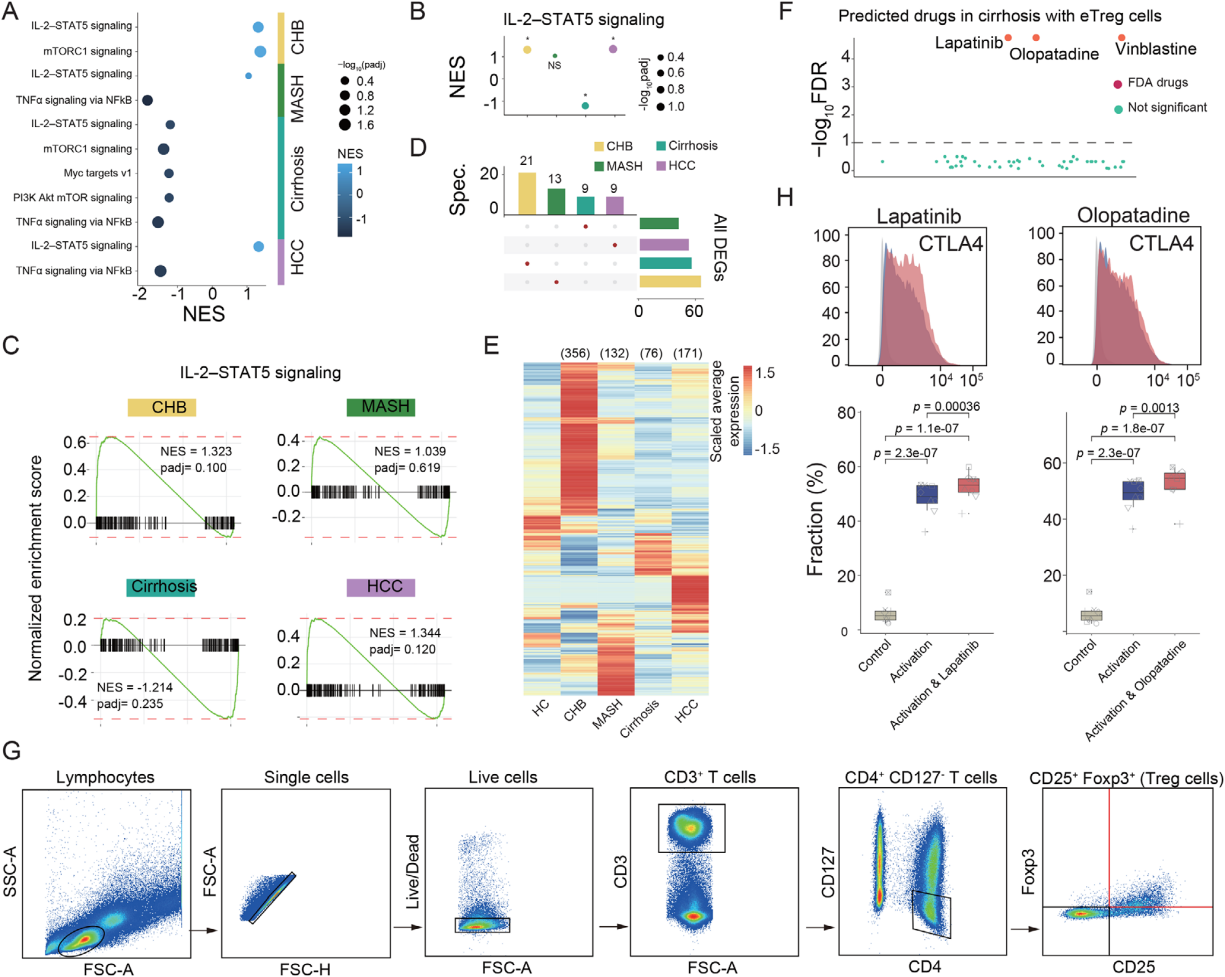
**Pathway signaling and drug repurposing on intrahepatic eTreg subpopulation**. (A) Functional enrichment results from differential hepatic disease individuals through differential expression analysis. (B) Normalized enrichment score (NES) of IL‐2–STAT5 signaling among differential hepatic disease individuals. Asterisks represent *p*‐adjusted less than 0.05, and NS represents not significant. (C) GSEA enrichment plots showing IL‐2–STAT5 signaling among differential hepatic disease individuals. (D) The number of DEGs enriched in IL‐2–STAT5 signaling among differential hepatic disease individuals. (E) Heat map showing the expression of DEGs of the eTreg subpopulation from CHB, MASH, cirrhosis, and HCC. The numbers in parentheses represent the number of DEGs. (F) Predicted drugs to reverse the expression patterns of the eTreg subpopulation in cirrhosis individuals. (G) The gating strategy for Treg cells. (H) Typical CTLA4 expression in Treg cells from PBMC of cirrhosis patients (*n* = 8) of unstimulated (gray), anti‐CD3/CD28 stimulated (blue) and 1 nM Lapatinib or Olopatadine intervention (red).

Simultaneously, the varied expression of intrahepatic eTreg subpopulation was also used as a target to predict putative drugs (Figures 6D, 7E, F, and S17A). A total of 596 DEGs in eTreg cells were estimated (Figures 6D and 7E), of which 76 DEGs were identified in cirrhosis patients through comparison to HC donors. Using ASGARD‐based drug prediction with DEGs, three FDA‐approved drugs, Lapatinib, Olopatadine, and Vinblastine, were predicted for cirrhosis (Figure 7F; Table S15). Lapatinib and Olopatadine significantly upregulated CTLA‐4 expression on Tregs from PBMC of cirrhosis patients (Figures 7G, H and S17B; Table S16), which is likely to reinforce Treg stability and suppressive capacity. These interventions also affected several biological pathways enriched in DEGs from the eTreg population of cirrhosis patients, such as TNFα and mTORC1 signals (Figure S17C).

Together, these results demonstrate that the disease‐specific cellular states defined in Pheat can serve as a powerful engine for discovering and validating novel immunotherapeutic agents for a range of hepatic diseases.

## 3 Discussion

In this study, we constructed Pheat, a comprehensive T‐cell atlas from large‐scale scRNA‐Seq data covering 139 individuals, 255 samples, and over 600 thousand cells. As a supplement to the human healthy liver cell atlas [35], Pheat integrates chronic hepatitis, cirrhosis, and HCC, to address the challenge of immunological heterogeneity across a spectrum of hepatic diseases. Pheat holds a high‐resolution T‐cell catalog categorized as CD4^+^ T, CD8^+^ T, pro T, and unconventional T‐cell subsets. A central insight is that both exhaustion and regulatory programs are not uniform but vary markedly with disease etiology and stage, a realization with significant implications for immunotherapy. This work provides not only novel biological insights but also a powerful, publicly accessible resource to accelerate future research in hepatic immunology.

A primary contribution of Pheat is the systematic deconstruction of T‐cell heterogeneity across cellular, tissue, and disease dimensions. At the population level, Pheat delineated canonical subsets comparable to prior atlas studies [28, 29, 30], but further revealed disease‐specific differences in Tex and Treg subpopulations [15, 36, 37, 38]. Consistent with previous single‐disease studies [51, 52], we observed that intrahepatic Tex cells were consistently enriched compared with PBMC, with Tex^pre^ cells depleted in CHB. This gradient was also evident along the HBV disease spectrum, from CHB to HBV^+^ cirrhosis to HBV^+^ HCC, and Tex^pre^ cells were more abundant in HBV^−^ than HBV^+^ HCC tumors, highlighting how chronic viral antigen exposure influences exhaustion trajectories. Frequencies of intrahepatic eTreg also exhibited the characteristic of dynamic change, generally increasing in hepatic diseases, but relatively rare in MASH and cirrhosis compared to CHB and HCC [53]. By integrating multiple hepatic diseases beyond HBV, Pheat extends these observations into a cross‐disease framework, enabling direct comparisons of T‐cell dysfunction shaped by viral, fibrotic, and malignant contexts.

Beyond cataloging cellular diversity, a key goal of Pheat was to identify actionable therapeutic targets. By focusing on the immunotherapy‐responsive Tex^pre^ population, we identified both established and novel disease‐specific immune checkpoints, which may be shaped by multiple disease‐specific factors, including the nature and duration of antigen exposure, inflammatory cytokine milieu, metabolic stress, stromal remodeling, and cellular interactions within the local tissue microenvironment [54, 55]. For example, 4‐1BB was preferentially expressed in CHB, and stimulation of this pathway has been shown to enhance cytokine production, such as IFN‐γ and TNF‐α, in PBMC from HBV‐infected patients. Moreover, 4‐1BB expression can be regulated by CREM activity, which is relatively low in the Tex^pre^ subset [42, 56, 57], suggesting a potential axis for therapeutic intervention. In contrast, TIGIT was preferentially expressed in HCC, consistent with its role in reinforcing T‐cell dysfunction in the tumor microenvironment and representing another disease‐specific checkpoint that could be therapeutically targeted [58].

In the Treg compartment, we found that IL‐2–STAT5 signaling was highly active in CHB and HCC but suppressed in cirrhosis. Given the essential role of IL‐2 in sustaining Treg suppressive function and metabolic fitness [48], these divergent patterns suggest different immunological pressures. In CHB and HCC, persistent antigen stimulation and chronic inflammation may enhance IL‐2 availability or Treg responsiveness, reinforcing their suppressive activity, whereas in cirrhosis, stromal remodeling and cytokine imbalance may limit IL‐2 production or signaling, reducing IL‐2–STAT5 activity [16, 48, 52, 59]. In this context, IL‐2 has attracted attention as a therapeutic target: modulation of IL‐2 can augment Treg activity in tumors [49], while CD8‐targeted IL‐2 muteins have been developed to activate dysfunctional CD8^+^ T cells and restore hepatic immunity in HBV models [50]. These differences imply that IL‐2–based interventions should be applied cautiously in CHB and HCC, but may hold therapeutic potential in cirrhosis.

For drug prediction, we focused on Tex and Treg subsets as therapeutic entry points [60]. By leveraging disease‐specific transcriptional signatures, Pheat facilitates the identification of candidate compounds across hepatic diseases, including several FDA‐approved agents. Among these, we experimentally confirmed that in CHB an HDAC inhibitor enhanced Tex^pre^ antiviral activity, but not that of Tex^term^, consistent with the model that the therapeutic effect is mediated by preventing the over‐activation of the T‐cell exhaustion program [61], while kinase‐ and histamine‐related drugs modulated Treg function in cirrhosis. More broadly, these examples illustrate the potential of Pheat as a resource for linking immune cell states to druggable pathways. Rather than being limited to a few candidate compounds, this framework offers a generalizable approach for rational drug repurposing and the design of precision immunotherapies in hepatic disease.

This study also has limitations. Firstly, we focused primarily on Tex and Treg populations, whereas other subsets such as MAIT, NKT, and γδ T cells also represent promising therapeutic targets [62]. Our analysis relied largely on publicly available datasets, limiting sample diversity and longitudinal resolution, especially liver tissue samples and some disease subgroups, particularly specific etiologies or disease stages within individual hepatic diseases, such as primary biliary cholangitis (PBC)‐related cirrhosis, which were underrepresented; this will be overcome to improve the robustness of comparative analyses in future studies. Secondly, the definition of T‐cell subpopulations, such as Tex^int^, Tex^term^, nTreg, and eTreg in this study, was based on transcriptomic signatures, which may not always directly correlate with protein expression due to post‐transcriptional and post‐translational regulation. In the future, incorporating protein‐level validation (e.g., flow cytometry or immunostaining) will be valuable to further confirm these findings. In addition, transcriptomic profiling alone cannot fully capture epigenetic, cytokine, metabolic, stroma, or spatial regulation of T‐cell function. Thirdly, in terms of mechanism depth and verification breadth, due to the accessibility limitations of liver tissues from hepatic diseases, we used PBMC samples to experimentally validate the candidate drugs (Panobinostat, Lapatinib, and Olopatadine) identified based on intrahepatic Tex^pre^ or eTreg cells in silico, which may not fully capture the sole hepatic immune microenvironment. Besides, incorporating humanized mouse models, organoid systems, and in vivo therapeutic testing will be required to establish the clinical translational potential of computationally predicted drugs and related interventions. Future studies should expand to additional hepatic conditions, integrate multi‐omics (epigenomic, proteomics and spatial technologies), and incorporate prospective clinical cohorts to validate therapeutic hypotheses.

In conclusion, Pheat provides a high‐resolution reference of hepatic T‐cell states across multiple diseases. By revealing disease‐specific programs of exhaustion and regulation, and linking them to therapeutic opportunities, this work contributes to a more nuanced framework for understanding hepatic immunity and offers a resource to inform precision immunotherapy development.

## 4 Methods and Materials

### 4.1 scRNA‐Seq Data Collection and Gene‐Cell Expression Abundance Generation

scRNA‐Seq data from differential hepatic diseases and HC donors were collected from public databases (Table S1, Supplementary materials), and then aligned and quantified with the 10× Genomics Cell Ranger v6.1.2 [63] against the human reference genome (hg38).

### 4.2 Processing and Integration of scRNA‐Seq Data

The doublet score of each cell was first estimated for each sample using Scrublet (version 0.2.3) [64]. Two rounds of scRNA‐Seq data integration, clustering and annotation were performed with the Seurat package (version 4.4.0) to construct the Pheat. For the first round, scRNA‐Seq processing was handled at the study level. In detail, high‐quality cells were extracted from each sample in each study according to the filtration criteria through UMI counts, gene counts, and mitochondrial gene fraction (Supplementary materials), and then integrated by Merge. Gene‐barcode matrices were normalized and scaled using the functions of NormalizeData and ScaleData, respectively. The top 2000 highly variable genes were detected by FindVariableFeatures. After principal component analysis (PCA) dimensionality reduction by runPCA and batch effect removal by RunHarmony (version 1.0.3) [65], uniform manifold approximation and projection (UMAP) coordinates of each cell were calculated. Immune cells were further clustered through FindNeighbors with the parameters at dims 1:30 and FindClusters function with a suitable resolution, leading to the identification of different immune cell populations. DEGs from each population were estimated by Findallmarker by comparing to other populations. Canonical marker genes and DEGs were used for assigning cell type for each cluster (T cells: *CD3D* ^+^, *CD3E* ^+^; NK cells: *KLRD1* ^+^, *GNLY* ^+^, *NKG7* ^+^; B cells: *MZB1* ^+^, *CD79A* ^+^; Plasma cells: *MZB1* ^+^, *CD38* ^+^; Myeloid cells: *LYZ* ^+^, *CD14* ^+^). To avoid cell noise, marker genes including *CD2*, *CD3E*, *CD3D*, and *CD3G* were further used for retaining T cells. Then, T cells from each study were integrated together for the second‐round annotation. Similar with the first round, all T cells were further clustered and annotated into CD4^+^ T (*CD3D* ^+^, *CD3E* ^+^, *CD4* ^+^), CD8^+^ T (*CD3D* ^+^, *CD3E* ^+^, *CD8A* ^+^, *CD8B* ^+^), MAIT (*CD3E* ^+^, *SLC4A10* ^+^), γδ T (*CD3D* ^+^, *TRDC* ^+^, *TRGC1* ^+^, *TRGC2* ^+^), NK T (*CD3E* ^+^, *KLRF1* ^+^, *XCL1* ^+^, *KLRC1* ^+^, *IL2RB* ^+^, *CD7* ^+^, *CCL3* ^+^), and pro T (*MKI67* ^+^, *PCNA* ^+^) cells.

### 4.3 Feature Analysis of Cell Populations

Functional scores of cell populations were calculated using AddModuleScore function of the Seurat package with gene signatures. Pseudotime trajectory analysis was performed using Slingshot (version 2.8.0) R package with default parameters [66]. Enrichment status of immune cell types across different tissues was estimated through Ro/e with chi‐square test. Fisher exact tests were performed and *p*‐values were adjusted using the Bonferroni method to evaluate the significance level.

### 4.4 Gene Set Enrichment Analysis

Gene set enrichment analysis (GSEA) was performed based on the fold‐change of genes with fgsea (version 1.26.0) [67]. The annotation information of gene sets was downloaded from MSigDB with the package msigdbr [68].

### 4.5 Differential Abundance Analysis

Differences in Tex abundance associated with HBV antigen load were tested using the R package Milo (version 2.9.1) [69] with default parameters.

### 4.6 TCR Analysis

Single‐cell TCR data of liver samples from CHB patients [70] were first assembled with the vdj pipeline from the 10× Genomics Cell Ranger v6.1.2 against Human V(D)J reference 5.0.0 (GRCh38), and then obtained high‐quality α–β pairs as previously described. TCR results were integrated with scRepertoire (version 2.9.0) [71]. Clonal expansion levels were evaluated with the function clonalBin. Cross‐T cluster transition among the three Tex subpopulations was analyzed by the STARTRAC pipeline (https://github.com/Japrin/STARTRAC) to elaborate the developmental trajectory through a high STARTRAC transition score between two clusters, indicating a developmental connection [72].

### 4.7 Co‐Expression Analysis

Co‐expression networks constructed by DEGs were built by GrnBoost2 from the module arboreto (version 0.1.6) [73]. Expression abundance of the Tex^pre^ subpopulation from different hepatic diseases was used as input to GrnBoost2, respectively, through default parameters. Disease‐specific co‐expression networks were characterized with nodes and edges, where a node was represented as a gene and an edge was represented as a relationship between two genes. We only retained edges connected to at least one ICG, and the top 2% edges sorted by weight were retained. The hub ICGs were identified through the number of edges, which was more than 10.

### 4.8 Drug Analysis

Putative drugs were predicted based on DEGs by the ASGARD package (version 1.0.0) [44]. Mono‐drugs repurposing for each disease was performed by the GetDrug with the parameters of drug.type set “all” indicating identification of both FDA‐approved drugs and compounds. Drug score was estimated by the function of DrugScore and then performed mono‐drug therapy selection by TopDrug with a false discovery rate < 0.1.

### 4.9 Flow Cytometry

Peripheral blood was collected in ethylenediaminetetraacetic acid‐anticoagulated tubes. PBMC samples were isolated using Ficoll‐Hypaque density‐gradient centrifugation. Isolated PBMC were stored in liquid nitrogen. PBMC were resuspended and incubated in RPMI 1640 containing 10% fetal bovine serum (FBS) at 37°C and 5% CO_2_ for 2 h. The Tex^pre^, Tex^term^, and Treg cells were detected as described in other studies [10, 74]. Briefly, surface markers were stained with monoclonal antibodies (mAbs) for 30 min at 4°C. For intracellular staining, samples were fixed and permeabilized using a Foxp3/transcription factor staining buffer set (Thermo Fisher Scientific) and stained intracellularly with the indicated antibodies for 30 min at 4°C. The stained samples were examined by flow cytometry on a BD Canto II flow cytometer (BD Biosciences). Flow cytometry data were analyzed using FlowJo software (version 10.5.3; BD Biosciences).

The following fluorescently conjugated antibodies were used for multicolor flow cytometry: APC/Fire 750 anti‐CD3 (SK7), PerCPcy5.5 anti‐CD8 (SK1), PE anti‐TCF1 (7F11A10), APC anti‐CD279 (A17188A), BV510 anti‐IFN‐g (4S.B3), FITC anti‐CD107a (H4A3), and PE anti‐FOXP3 (259D) from BioLegend; FITC anti‐CD4 (RPA‐T4), APC anti‐CD25 (M‐A251), PerCPcy5.5 anti‐CD45 (HI30), BV510 anti‐CD127 (HIL‐7R‐M21), BV421 anti‐CD152 (BNI3), and Fixable Viability Stain 700 from BD Biosciences.

### 4.10 *Ex Vivo* Examination of the Effects of Candidate Drugs on Tex^pre^, Tex^term^, and Treg Cell Function

To assess the functional properties of Tex^pre^ and Tex^term^ cells, PBMC were stimulated with anti‐CD3 antibody (1 µg/mL) and anti‐CD28 antibody (1 µg/mL) for 48 h, an additional phorbol 12‐myristate 13‐acetate (50 ng/mL) and ionomycin (1 µg/mL) was given 6 h prior to the termination of the intervention, synchronized addition of drug candidates from the start of the intervention. To detect the function of Treg cells, PBMCs were stimulated with anti‐CD3 (1 µg/mL) and anti‐CD28 (1 µg/mL) for 48 h, and the candidate drugs were added simultaneously. Golgi stop was added to the medium 6 h before harvest to detect intracellular cytokines. The concentrations of candidate drugs used in the experiments were as follows: Panobinostat, 1 nM; Lapatinib, 1 nM; Olopatadine, 1 nM.

The following reagents were used for *ex vivo* examination: CD3 Monoclonal Antibody (OKT3), Functional Grade (16‐0037‐81, Thermo Fisher Scientific), CD28 Monoclonal Antibody (CD28.2), Functional Grade (16‐0289‐81, Thermo Fisher Scientific), Cell Stimulation Cocktail (00‐4970, Thermo Fisher Scientific), GolgiStop Protein Transport Inhibitor (554724, BD Biosciences), Panobinostat (HY‐10224, MCE), Lapatinib (HY‐50898, MCE), Olopatadine hydrochloride (HY‐B0426A, MCE).

### 4.11 Multiplex Immunofluorescent Assays

Multiple immunofluorescent staining of liver tissues was performed as previously described [75]. Briefly, FFPE liver tissue sections were baked for 2 h at 60°C before staining. Slides were rehydrated with a series of graded ethanol solutions in deionized water. Antigen retrieval was performed at pH 6 for 20 min at 95°C. Slides were serially stained with the following antibodies: anti‐CD8, ‐PD1, ‐TCF1, ‐TIGIT and ‐4‐1BB. Anti‐rabbit horseradish peroxidase (Cell Signaling Technology) was used as the secondary antibody with an incubation time of 10 min. TSA‐conjugated fluorophores (PerkinElmer) were used to visualize each biomarker, consisting of 690 (CD8), 520 (PD1), 570 (TCF1), 620 (TIGIT) and 480 (4‐1BB), with incubation times for each primary antibody being 1 h. Slides were mounted with anti‐fade mounting medium (Life Technologies) and stored at 4°C before imaging. Images of whole slides were captured (40× magnification for multispectral images) using the phenoimager HT 2.0 multispectral imaging platform (Akoya Biosciences), and the images were analyzed using the inForm Tissue Analysis Software (Akoya Biosciences), with cell percentages calculated and locations labeled.

### 4.12 Statistical Analysis

Statistical analysis of *ex vivo* examination results was performed using the function stat_compare_means in the ggpubr R package with the parameters set as method = t.test and paired = T.

## Author Contributions

CZ and FSW were involved in the project's initial conceptualization and experimental study design. SYC conducted scRNA‐Seq analysis. YYW planned and performed experiments. SNZ was involved in the sample collection. SYC and CZ wrote the manuscript with critical inputs from all authors, including MJZ, XL, WZL, MMZ, YXY, YS, PF, JL, XF, RNX, YMJ, JWS, CZ, and MS. All authors have read and approved the final manuscript.

## Funding

This work was supported by the National Science and Technology Major Project (2025ZD01905602), the National Key Research and Development Program of China (2022YFA1303600 and 2023YFC2308100), the National Natural Science Foundation of China (82130019 and 82272311), the Capital Funds for Health Improvement and Research (2024‐1‐2182), and the Beijing Nova Program (20240484606).

## Ethics Statement

The single‐cell transcriptomic analyses in this study were based on publicly available, de‐identified datasets, for which ethical approvals and informed consent were addressed in the respective original studies. No additional participant recruitment was undertaken for these secondary analyses.

The experimental validation involving patient‐derived peripheral blood mononuclear cells (PBMCs) and liver tissue specimens, including multiplex immunofluorescence staining and *ex vivo* drug treatment experiments, was approved by the Ethics Committee of the Fifth Medical Center of the Chinese PLA General Hospital (approval number KY‐ZDZX‐2025‐11‐215‐1). Written informed consent was obtained from the participants who provided specimens for these validation experiments, in accordance with the Declaration of Helsinki.

## Conflicts of Interest

All authors declare no competing interests.

## Supporting information




**Supporting File 1**: mco271146‐sup‐0001‐TableS1.xlsx

**Supporting File 2**: mco271146‐sup‐0002‐TableS2.xlsx

**Supporting File 3**: mco271146‐sup‐0003‐TableS3.xlsx

**Supporting File 4**: mco271146‐sup‐0004‐TableS4.xlsx

**Supporting File 5**: mco271146‐sup‐0005‐TableS5.xlsx

**Supporting File 6**: mco271146‐sup‐0006‐TableS6.xlsx

**Supporting File 7**: mco271146‐sup‐0007‐TableS7.xlsx

**Supporting File 8**: mco271146‐sup‐0008‐TableS8.xlsx

**Supporting File 9**: mco271146‐sup‐0009‐TableS9.xlsx

**Supporting File 10**: mco271146‐sup‐0010‐TableS10.xlsx

**Supporting File 11**: mco271146‐sup‐0011‐TableS11.xlsx

**Supporting File 12**: mco271146‐sup‐0012‐TableS12.xlsx

**Supporting File 13**: mco271146‐sup‐0013‐TableS13.xlsx

**Supporting File 14**: mco271146‐sup‐0014‐TableS14.xlsx

**Supporting File 15**: mco271146‐sup‐0015‐TableS15.xlsx

**Supporting File 16**: mco271146‐sup‐0016‐TableS16.xlsx

**Supporting File 17**: mco271146‐sup‐0017‐SuppMat.docx

## Data Availability

A detailed description of scRNA‐Seq datasets utilized in this study, including data sources and accession numbers of the scRNA‐Seq datasets, is provided in Supplementary Materials. Pheat web portal can be accessed at http://211.166.247.226/ncrc/infectious/xxdt/detail/12693.html, and its user manual is available from GitHub (https://github.com/chenzhuod/Pheat/).
