# Supplementary material for: A Pan‐Hepatic T‐Cell Atlas Identifies Disease‐Specific Immune Dysfunction and Therapeutic Targets for Immunotherapy
Source: MedComm (2020). 2026 Nov 8;7(11):e71146. doi: 10.1002/mco2.71146 (PMC13744652; doi:10.1002/mco2.71146)
Supplement: Supplementary file 1 [file MCO2-7-e71146-s004.docx]

***Supplementary Materials***

**Data collection and pre-processing**

In this study, we integrated 255 scRNA-Seq datasets from patients with differential hepatic diseases and healthy controls (HC) from 12 hepatic disease-related studies. Data from HBV infection patients and corresponding HC were sequenced by our team. Data from other hepatic diseases and corresponding HC donors were downloaded from public databases, including the Genome Sequence Archive (GSA), Sequence Read Archive (SRA) database, and Gene Expression Omnibus (GEO) through searching the keywords "'scRNA-Seq' and 'HCV'", "'scRNA-Seq' and 'Alcohol‐associated hepatitis'", "'scRNA-Seq' and 'Metabolic dysfunction-associated steatohepatitis'", "'scRNA-Seq' and 'Cirrhosis'", "'scRNA-Seq' and 'HCC'" as of 9 July 2024.

**1. HBV-infection patients and corresponding HC donors**

47 liver biopsies and PBMC scRNA-Seq samples from 19 differential phase HBV-infected patients and 6 healthy donors were enrolled. Concretely, the phases of HBV-infection patients include immune tolerant (IT, 6 individuals), immune active (IA, 6 individuals), acute hepatitis B (AHB, 4 individuals) and chronic resolved (CR, 3 individuals) phases ^1^. Underwent a Cellranger (10x Genomics Cell Ranger v6.1.2) and Seurat (version 4.4.0) pipeline analysis, high-quality cells were kept after quality control based on a gene appearing in at least 3 cells, a cell with 600~10,000 genes and less than 10% mitochondrial gene counts.

**2. HCV-infection patients and corresponding HC donors**

CD3^+^ T-sorted intrahepatic scRNA-Seq data from six chronic hepatic C patients before, during at 4 weeks, and after treatment at 12 weeks with ledipasvir/sofosbuvir, respectively, and three healthy donors were downloaded from the GEO database (GSE178756) ^2^. After Cellranger (10x Genomics Cell Ranger v6.1.2) for quantity and Seurat (version 4.4.0) for integration pipeline analysis, quality control was implemented by a criterion of retaining cells with either more than 300 genes detected or fewer than 3,500 genes, and with less than 10% of mitochondrial gene counts to generate high-quality cells.

**3. Alcohol-related hepatitis patients and corresponding HC donors**

scRNA-Seq data of PBMCs from four alcohol-related hepatitis patients and four age-matched healthy controls were collected from the GEO database (PRJNA596980) ^3^. High-quality cells were obtained with cells either having more than 200 genes detected or fewer than 4,000 genes, and with less than 20% of mitochondrial gene counts.

**4. Metabolic Dysfunction-Associated Steatohepatitis and corresponding HC donors**

Six MASH (referred to as non-alcoholic steatohepatitis) patients and four paired healthy individuals were enrolled for single-cell RNA sequencing ^4^. Sequencing read data were downloaded from the GEO database (GSE144635). After read-genome alignment with Cellranger, the gene-barcode matrix of each sample was input to Seurat to filter out low-quality cells with fewer than 200 genes or more than 6,000 genes detected per cell, and more than 10% mitochondrial genes per cell to generate high-quality cells.

**5. Cirrhosis patients and corresponding HC donors**

(i) Two healthy donors and two patients diagnosed with HBV cirrhosis were enrolled and single-cell RNA sequencing was performed on the liver tissue ^5^. High-quality cells were obtained based on a gene appearing in at least 3 cells, a cell with 600~10,000 genes and less than 10% of mitochondrial gene counts (PRJNA833766 from the SRA database).

(ii) 24 scRNA-Seq data were obtained from liver and PBMC samples from GEO databases (GSE136103), of which the liver samples were from five cirrhotic patients and five healthy donors, and the PBMC samples were from four cirrhotic patients^6^. High-quality cells were retrieved from all scRNA-Seq reads with a criterion of excluding cells with fewer than 300 expressed genes and more than 30% mitochondrial gene content. Genes expressed in at least three cells in a sample were retained.

(iii) Liver samples from 12 patients were collected, including 7 non-cirrhotic and 5 cirrhotic patients. scRNA-Seq data of a total of 33 samples from these 12 donors were downloaded from the GEO database (GSE168933)^7^. Raw sequencing data were mapped to the GRCh38 human reference genome with 10x Genomics Cell Ranger 6.1.2. Quality control was further performed on gene-barcode count matrices with the criteria of more than 800 UMI counts per cell and less than 20% mitochondrial transcripts per cell to generate high-quality cells.

**6. HCC patients and corresponding HC donors**

(i) Liver tissue from eight HCC patients was sequenced to generate scRNA-Seq data ^8^. Quality control of raw data was performed by retaining cells with 600~10,000 genes and less than 10% of mitochondrial gene counts (SRP318499 from the SRA database).

(ii) Seven pairs of HCC tumors and non-tumor liver tissues were used to perform single-cell RNA sequencing ^9^. After quantifying gene expression abundance, gene-barcode metrics were input into Seurat to perform quality control with the criteria of retaining a cell with 800~2,000 genes and less than 20% of mitochondrial gene counts (CRA002308 from the GSA database).

(iii) A total of 20 HCC liver tissue samples, including tumor and adjacent tissue, were used for single-cell RNA sequencing ^10^. High-quality cells were obtained from all scRNA-Seq reads with a criterion of excluding cells with fewer than 500 expressed genes and more than 10% mitochondrial gene content (GSE189903 from the GEO database).

(iv) High-quality cells were collected from 14 HCC patients and one healthy control donor by excluding cells with fewer than 200 expressed genes and fewer than 5% mitochondrial gene content ^11^ (GSE156337 from the GEO database).

(v) Seven tumors from patients with untreated HBV-related HCC and adjacent liver tissues from four of these patients were enrolled in this study ^12^. High-quality cells were generated through the criteria of cells with fewer than 200 expressed genes and fewer than 10% mitochondrial gene content (GSE202642 from the GEO database).

**Reference**

1.	Zhang C, Li J, Cheng Y, et al. Single-cell RNA sequencing reveals intrahepatic and peripheral immune characteristics related to disease phases in HBV-infected patients. *Gut*. Jan 2023;72(1):153-167. doi:10.1136/gutjnl-2021-325915

2.	Burchill MA, Salomon MP, Golden-Mason L, et al. Single-cell transcriptomic analyses of T cells in chronic HCV-infected patients dominated by DAA-induced interferon signaling changes. *PLoS Pathog*. Aug 2021;17(8):e1009799. doi:10.1371/journal.ppat.1009799

3.	Kim A, Bellar A, McMullen MR, Li X, Nagy LE. Functionally diverse inflammatory responses in peripheral and liver monocytes in alcohol-associated hepatitis. *Hepatol Commun*. Oct 2020;4(10):1459-1476. doi:10.1002/hep4.1563

4.	Pfister D, Nunez NG, Pinyol R, et al. NASH limits anti-tumour surveillance in immunotherapy-treated HCC. *Nature*. Apr 2021;592(7854):450-456. doi:10.1038/s41586-021-03362-0

5.	Bai Q, Hong X, Lin H, et al. Single-cell landscape of immune cells in human livers affected by HBV-related cirrhosis. *JHEP Rep*. Nov 2023;5(11):100883. doi:10.1016/j.jhepr.2023.100883

6.	Ramachandran P, Dobie R, Wilson-Kanamori JR, et al. Resolving the fibrotic niche of human liver cirrhosis at single-cell level. *Nature*. Nov 2019;575(7783):512-518. doi:10.1038/s41586-019-1631-3

7.	Buonomo EL, Mei S, Guinn SR, et al. Liver stromal cells restrict macrophage maturation and stromal IL-6 limits the differentiation of cirrhosis-linked macrophages. *J Hepatol*. May 2022;76(5):1127-1137. doi:10.1016/j.jhep.2021.12.036

8.	Ho DW, Tsui YM, Chan LK, et al. Single-cell RNA sequencing shows the immunosuppressive landscape and tumor heterogeneity of HBV-associated hepatocellular carcinoma. *Nat Commun*. Jun 17 2021;12(1):3684. doi:10.1038/s41467-021-24010-1

9.	Song G, Shi Y, Zhang M, et al. Global immune characterization of HBV/HCV-related hepatocellular carcinoma identifies macrophage and T-cell subsets associated with disease progression. *Cell Discov*. Dec 8 2020;6(1):90. doi:10.1038/s41421-020-00214-5

10.	Ma L, Heinrich S, Wang L, et al. Multiregional single-cell dissection of tumor and immune cells reveals stable lock-and-key features in liver cancer. *Nat Commun*. Dec 7 2022;13(1):7533. doi:10.1038/s41467-022-35291-5

11.	Sharma A, Seow JJW, Dutertre CA, et al. Onco-fetal reprogramming of endothelial cells drives immunosuppressive macrophages in hepatocellular carcinoma. *Cell*. Oct 15 2020;183(2):377-394 e21. doi:10.1016/j.cell.2020.08.040

12.	Zhu GQ, Tang Z, Huang R, et al. CD36^+^ cancer-associated fibroblasts provide immunosuppressive microenvironment for hepatocellular carcinoma via secretion of macrophage migration inhibitory factor. *Cell Discov*. Mar 6 2023;9(1):25. doi:10.1038/s41421-023-00529-z

13.	Chu Y, Dai E, Li Y, et al. Pan-cancer T cell atlas links a cellular stress response state to immunotherapy resistance. *Nat Med*. Jun 2023;29(6):1550-1562. doi:10.1038/s41591-023-02371-y

**Figure S1 UMAP visualization showing the harmony-based batch-effect correction result with before (A) and after (B) correction.**

**Figure S2** An interactive web portal of Pheat providing open access to gene expression data, cell annotations, and visualization tools.

**Figure S3 Transcriptional heterogeneity of CD8^+^** **T cells.** (A) Bar plot showing the distribution of five CD8^+^ T cell populations across differential hepatic diseases. Abbreviations of diseases are in line with Figure 1. (B) Expression of ten gene signatures (as listed in Table S2, collected from ^13^) across CD8^+^ T cell clusters. (C-D) UMAP (C) and box plot (D) showing the frequency difference of five CD8^+^ T cell populations in PBMC and liver tissue. (E) Box plot showing the cellular proportion of five CD8^+^ T cell populations across differential hepatic diseases in PBMC (left) and liver (right).

**Figure S4 Transcriptional heterogeneity of CD4^+^** **T cells.** (A) Bar plot showing the distribution of five CD4^+^ T cell populations across differential hepatic diseases. Abbreviations of diseases are in line with Figure 1 (B) Expression of eight gene signatures (as listed in Table S2, collected from^13^) across CD4^+^ T cell subsets. (C-D) UMAP (C) and box plot (D) showing the frequency difference of five CD4^+^ T cell populations in PBMC and liver tissue. (E) Box plot showing the cellular proportion of five CD4^+^ T cell populations across differential hepatic diseases in PBMC (left) and liver (right).

**Figure S5 Landscape of proliferative T and unconventional T cells.** (A) UMAP showing three proliferative T cell clusters, including CD4-MTIX, CD8-GZMK, and CD8-CLL4. (B) Bubble plot showing marker gene expression across differential proliferative T clusters. (C) Distribution of proliferative T clusters across PBMC and liver tissue. Bar plot (upper) showing the relative proportion of cells for each proliferative T cell type in PBMC and liver tissue. Heat map (lower) displaying tissue prevalence estimated by Ro/e. (D-E) Cellular proportion of five proliferative T cell states across individuals with differential hepatic diseases and HC donors in PBMC (D) and liver tissue (E), respectively. (F) UMAP showing five unconventional T cells: MAIT, γδ T-1, γδ T-2, NKT-GZMB, and NKT-TIGIT. (G) Bubble plot showing marker gene expression across differential unconventional T clusters. (H) Distribution of unconventional T clusters across PBMC and liver tissue. Bar plot (upper) showing the relative proportion of cells for each unconventional T cell type in PBMC and liver tissue. Heat map (lower) displaying tissue prevalence estimated by Ro/e. (I-J) Cellular proportion of five unconventional T cell states across individuals with differential hepatic diseases and HC donors in PBMC (I) and liver tissue (J), respectively.

**Figure S6 Expression abundance and biological function of Tex cells.** (A) UMAP shows the expression of exhaustion markers, including *TOX*, *TIGIT*, *CTLA4*, and *TNFRSF9* in the Tex population. (B) GSEA enrichment plots showing the function of differential Tex subpopulations.

**Figure S7 Developmental trajectory analysis for Tex subpopulations.** (A) Pseudotime analysis for Tex subpopulations using Slingshot. (B) Milo beeswarm plot showing the differential abundance of neighbourhoods in CHB patients across HBV antigen load, annotated by Tex cells. Red and blue neighbourhoods are significantly enriched in low or high HBV level, respectively. (C) The number of unique clones in each liver sample from CHB individuals. (D) UMAP showing the clonal size of each Tex cell. (E) Frequency of cells by six clonal-size clusters categorized into different clonal frequency ranges. (F) Developmental path inferred by STARTRAC using scTCR-Seq data. (G) Activity of transcriptional factors in differential Tex subpopulations.

**Figure S8 Frequency variance of the intrahepatic Tex subpopulations across differential hepatic diseases and HC donors.** (A) Box plot showing the cellular proportion of the three intrahepatic Tex subpopulations across differential hepatic disease individuals and HC donors. (B-C) Box plot showing the cellular proportion of the three intrahepatic Tex subpopulations across differential-phase HBV-infected (B) and HCC (C) patients compared to HC donors.

**Figure S9 Dynamics of cellular and molecular features of the Tex populations along the hepatitis trilogy.** (A) The proportion of three intrahepatic Tex subpopulations from healthy liver to CHB, HBV^+^ cirrhosis, and HBV^+^ HCC. (B-C) Distribution of DEGs along the hepatitis trilogy across differential Tex subpopulations. The red number represents the number of up-regulated DEGs, and the blue number represents the number of down-regulated DEGs. The red gene names represent up-regulated ICGs, and the blue gene names represent down-regulated ICGs.

**Figure S10 Dynamics of cellular and molecular features of the Tex populations between HBV^+^ HCC and HBV^-^ HCC.** (A) The proportion of Tex subpopulations across HBV^+^ HCC and HBV^-^ HCC individuals in the tumor and adjacent tissues, respectively. (B-C) Distribution of DEGs between HBV^+^ HCC and HBV^-^ HCC compared to HC donors across differential Tex subpopulations in tumor (B) and tumor adjacent tissue (C), respectively. The red number represents the number of up-regulated DEGs, and the blue number represents the number of down-regulated DEGs. The red gene names represent up-regulated ICGs, and the blue gene names represent down-regulated ICGs.

**Figure S11 Co-expression networks of DEGs and ICGs in MASH (A) and cirrhotic (B) patients.**

**Figure S12 Drug discovery through DEGs from the Tex^pre^ subpopulation.** (A) Multiplexed immunofluorescent staining of the Tex^pre^ cells marker panels (DAPI-blue, CD8-red, TCF1-yellow, PD1-green, TIGIT-orange and 4-1BB-light blue) of liver tissue in the HC group (40×, scale bars = 20 μm). (B) Drugs and compounds to reverse expression patterns of the Tex^pre^ subpopulation in MASH, cirrhosis, and HCC discovered through ASGARD. (C) Panobinostat treatment impacting multiple pathways, including the interferon γ response pathway and mTORC1 signaling. (D) Expression of interferon γ response signatures across CHB, AHB and CR patients. (E-F) Venn (E) and dot (F) plot showing the number and fold change (FC) of DEGs in the intrahepatic Tex^pre^ subpopulation from CHB, AHB and CR patients compared to HC donors. (G) Normalized enrichment score (NES) of interferon γ response pathway among CHB, AHB and CR patients. (H) Fraction of cell viability in Tex^pre^ cells using PBMC from CHB patients (n=3) of anti-CD3/CD28 and PMA/Iono stimulated (blue), and 1 nm Panobinostat intervention (red). (I) Representative IFN-γ expression in TCF1⁻PD-1⁺ terminally exhausted T cells using PBMC from CHB patients (n=6) of unstimulated (gray), anti-CD3/CD28 and PMA/Iono stimulated (blue), and 1 nm Panobinostat intervention (red).

**Figure S13 Expression features of the Treg subpopulations.** (A) Bubble plot showing expression of marker genes across two Treg subpopulations. (B) UMAP shows the expression of Treg subpopulation markers, including *CCR7*, *SELL*, *IL7R*, and *CTLA4,* in the Treg population.

**Figure S14 Functional enrichment results of the eTreg subpopulations from individuals with CHB (A-B), MASH (C-D), Cirrhosis (E-F), and HCC (G-H).**

**Figure S15 Dynamics of cellular and molecular features of the Treg populations along the hepatitis trilogy.** (A) The proportion of two Treg subpopulations from healthy liver to CHB, HBV^+^ cirrhosis, and HBV^+^ HCC. (B-C) Distribution of DEGs along the hepatitis trilogy across differential Treg subpopulations. The red number represents the number of up-regulated DEGs, and the blue number represents the number of down-regulated DEGs. The red gene names represent up-regulated ICGs, and the blue gene names represent down-regulated ICGs.

**Figure S16 Dynamics of cellular and molecular features of the Treg populations between HBV^+^ HCC and HBV^-^ HCC.** (A) The proportion of Treg subpopulations across HBV^+^ HCC and HBV^-^ HCC individuals in the tumor tissues. (B) Distribution of DEGs between the tumor tissue of HBV^+^ HCC and HBV^-^ HCC across differential Treg subpopulations. The red number represents the number of up-regulated DEGs, and the blue number represents the number of down-regulated DEGs. The red gene names represent up-regulated ICGs, and the blue gene names represent down-regulated ICGs.

**Figure S17 Drug discovery through DEGs from the eTreg subpopulation.** (A) Drugs and compounds to reverse expression patterns of the eTreg subpopulation in CHB, MASH, and HCC were discovered through ASGARD. (B) Fraction of cell viability in Treg cells from PBMC of cirrhosis patients (n=4) of anti-CD3/CD28 and PMA/Iono stimulated (blue), 1 nm Lapatinib intervention (red), and 1 nm Olopatadine intervention (yellow). (C) Lapatinib and Olopatadine treatment impacts multiple pathways.

**Table S1 List of scRNA-seq data**

**Table S2 Molecular signatures used for functional score estimation of CD8^+^ and CD4^+^ T cell subpopulations**

**Table S3 Molecular signatures used for functional score estimation of Tex subpopulations**

**Table S4 List and functional enrichment results of DEGs in different Tex subpopulations**

**Table S5 List of DEGs along the developmental trajectory of Tex**

**Table S6 List of DEGs along the hepatitis trilogy**

**Table S7 Cluster and expression abundance of ICGs**

**Table S8 List of DEGs in Tex^pre^ across different hepatic diseases**

**Table S9 List of predicted drugs through Tex^pre^ subpopulation**

**Table S10 Functional enrichment results of DEGs in Tex^pre^ across different hepatic diseases**

**Table S11 Clinical data of CHB patients used in experiment**

**Table S12 List of DEGs in eTreg across different hepatic diseases**

**Table S13 Functional enrichment results of DEGs in eTreg across different hepatic diseases**

**Table S14 List of DEGs in eTreg enriched in IL-2–STAT5 signaling**

**Table S15 List of predicted drugs through eTreg subpopulation**

**Table S16 Clinical data of cirrhotic patients used in the experiment**
